# Hypersensitivity reactions related to oxaliplatin (OHP)

**DOI:** 10.1038/sj.bjc.6601155

**Published:** 2003-07-29

**Authors:** G Brandi, M A Pantaleo, C Galli, A Falcone, A Antonuzzo, P Mordenti, M C Di Marco, G Biasco

**Affiliations:** 1 Institute of Haematology and Medical Oncology ‘L.& A. Seragnoli’, University of Bologna, Policlinico Sant'Orsola, Via Massarenti 9, 40138 Bologna, Italy; 2 Division of Medical Oncology, Department of Oncology, Civil Hospital, Livorno Viale Alfieri 36, 58128 Livorno, Italy

**Keywords:** Hypersensitivity reaction, platinum compound, colorectal cancer, chemotherapy

## Abstract

Patients treated with platinum compounds are subject to hypersensitivity reactions. Our study has highlighted the reactions related to oxaliplatin (OHP) infusion. One hundred and twenty-four patients affected by advanced colorectal cancer were treated with different schedules containing OHP, at the Institute of Haematology and Medical Oncology ‘L. and A. Seragnoli’ of Bologna and at the Medical Oncology Division of Livorno Hospital. Seventeen patients (13%) showed hypersensitivity reactions after a few minutes from the start of the OHP infusion. Usually, these reactions were seen after 2–17 exposures to OHP (Mean±s.e.: 9.4±1.07). No patient experienced allergic reactions at his/her first OHP infusion. Eight patients developed a mild reaction consisting of flushing and swelling of the face and hands, itching, sweating and lachrymation. The remaining nine patients showed a moderate–severe reaction with dyspnoea, wheezing, laryngospasm, psycho-motor agitation, tachycardia, precordial pain, diffuse erythema, itching and sweating. Six patients out of 17 were re-exposed to the drug with premedication of steroids and all except one developed the hypersensitivity reaction again. The cumulative dose, the time of exposure to OHP and the clinical features are variable and unpredictable. The risk of developing hypersensitivity reactions in patients treated with a short infusion of OHP cannot be underestimated.

Oxaliplatin (OHP) is the most recent platinum compound entering the clinical practice. It is an alkylating agent on DNA and forms DACH-platinum DNA adducts more hydrophobic than those formed by cisplatin (CDDP) and carboplatin (CBDCA). It is effective in advanced colorectal cancer both as a first-line therapy and in 5-fluorouracil (5-FU) refractory patients (Bertheault-Cvitkovic *et al*, 1996; De Gramont *et al*, 1997; Andre' *et al*, 1999; Maindrault-Goebel *et al*, 1999).

OHP is less nephro-ototoxic than CDDP and less mielotoxic than CBDCA (Misset 1998). The most characteristic and dose-limiting toxicity of OHP is sensory neuropathy, which is dose cumulative and schedule related. It is clinically characterised by a transient acute cold-related dysaesthesias, sometimes pain-associated, or with cramps and functional failure, although it is generally reversible (Caussanel *et al*, 1990, Misset, 1998). Hypersensitivity reactions to oxaliplatin have been described only sporadically.

For other platinum compounds, this kind of reaction is well known (Cleare *et al*, 1976; Wiesenfeld *et al*, 1979; Planner *et al*, 1991; Morgan *et al*, 1994; Weideman *et al*, 1994; Shleback *et al*, 1995; Markman *et al*, 1999; Özgüroglu *et al*, 1999). On data sheets of OHP, these clinical features are not stressed. In fact, only the main severe form of hypersensitivity, that is to say anaphylaxis, is reported in 0.5% of patients treated. This reaction is clinically characterised by laryngospasm and wheezing and immunologically linked to the release of histamine and other vaso-active substances.

As a result of the increasing use of OHP in colorectal cancer, we have found frequent hypersensitivity reactions. In this study, we report the epidemiological and clinical features of these reactions, as well as their management.

## MATERIALS AND METHODS

From February 1999 to February 2002 at the Institute of Haematology and Medical Oncology ‘L. and A. Seragnoli’ of Bologna and at the Medical Oncology Division of Livorno, 124 outpatients with advanced colorectal cancer were treated with OHP-based therapies. Eighty-four out of 124 patients (67.7%) received OHP as a first-line treatment. Fifty-five patients (44.3%) were treated with a FOLFOX-4 regimen (Andre' *et al*, 1999; De Gramont *et al*, 2000), 34 patients (27.4%) with FOLFOX-3 regimen (De Gramont *et al*, 1999), 30 patients (24.1%) with the association of OHP/CPT-11/c.i.5-FU/FA regimen (Falcone *et al*, 2002), three patients (2.4%) with OHP alone (Diaz-Rubio *et al*, 1998) and two patients (1.6%) with OHP/Raltitrexed regimen (Seitz *et al*, 1999). All patients received a standard antiemetic treatment with ondansetron 8 mg by a i.v. administration before chemotherapy. We did not use dexamethasone in this population.

Major sites of metastases were the liver, lungs and peritoneum. Among these patients, 17 out of 124 (13.7%) reported a hypersensitivity reaction attributable to OHP. There were eight males and nine female patients, with a mean age of 60.3 years (range 37–76). In 11 out of 17 patients with hypersensitivity reaction, OHP was administered in first-line chemotherapy.

## RESULTS

Results are shown in Table 1

**Table 1:** Patients with hypersensitivity reactions

**Case**	**Sites of metastases**	**Chemotherapy regimen**	**Infusion number at reaction**	**Total dose of OHP (mg)**	**Clinical features of reaction**	**Length of reaction (min)**	**Treatment of reaction**	**Re-exposure to OHP with premedication and outcome**
1	Lung	FOLFOX-4	3	382	Bronchospasm	7 days	Hospitalisation/high dose of steroid	No
					Laryngospasm			
					Dyspnoea			
2	Peritoneum	FOLFOX-4	14	2100	Bronchospasm	60	Oxygen	No
					Dyspnoea		Steroids	
					Hand oedema		Antihistaminic	
					Eriythema			
3	Liver	Oxaliplatin	10	2070	Bronchospasm	50	Steroids	No
					Dyspnoea			
					Hypotension			
4	Liver	FOLFOX-3	17	2271	Dyspnoea	5–10	Steroids	Yes, with reaction
	Lung				Hand, face oedema		Antihistaminic	
					Erythema, itching			
					Psychomotor agitation			
5	Peritoneum	Oxalipatin	2	360	Dyspnoea	120	Steroids	No
					Laryngospasm		Antihistaminic	
6	Liver	Irinotecan	11	1620	Dyspnoea	15–20	Steroids	No
		Oxaliplatin			Eye oedema		Antihistaminic	
		Fluorouracil			Face erythema			
		Folinic acid			Itching			
					Sweating			
7	Liver	FOLFOX-3	17	2720	Dyspnoea	15–20	Steroids	Yes, with reaction
	Lung				Oedema		Antihistaminic	
					Erythema			
					Sweating			
					Lachrymation			
8	Liver	FOLFOX-4	6	900	Dyspnoea	30	Steroids	No
					Erythema		Antihistaminic	
					Itching			
					Mouth oedema			
9	Liver	FOLFOX-4	8	1120	Dyspnoea	60	Steroids	No
	Lung				Hand, face erythema		Antihistaminic	
10	Liver	FOLFOX-4	8	1360	Erythema,	15–20	Steroids	No
					Tachycardia		Antihistaminic	
					Precordial pain			
					Pruritus			
11	Peritoneum	FOLFOX-4	9	630	Hand oedema, Hand genital itching	20	Antihistaminic	Yes, without reaction
					Hand, face erythema			
12	Peritoneum	FOLFOX-4	5	940	Hand face erythema	20–30	Antihistaminic	No
					Hand oedema			
					Hand itching			
13	Liver	Irinotecan	14	2600	Itching	15–20	Steroids	Yes, with reaction
	Peritoneum	Oxaliplatin			Sweating		Anthistaminic	
		Fluorouracil			Lachrymation			
		Folinic acid			Face oedema,			
					Face erythema			
14	Liver	FOLFOX-4	7	1040	Face, chest erythema	120	Steroids	Yes, with reaction
					Itching		Antihistaminic	
15	Liver	FOLFOX-4	13	1705	Face, chest erythema	50	Steroids	No
	Peritoneum				Shiver without fever		Antihistaminic	
					Tremor			
16	Diaphragm	FOLFOX-4	8	1080	Arms, chest erythema	50	Steroids	Yes, with reaction
					With pomphoid reaction			
17	Peritoneum	FOLFOX-3	9	1377	Sweating	15–20	Steroids	No
					Erythema			
					Hypotension			
					Nausea			

. The reaction occurs after a mean±s.e.=9.4±1.07 infusions of chemotherapy (range 2–17). Only two patients experienced early hypersensitivity at the second and third infusion, respectively.

On average, there were 217.7±32.5 days (mean±s.e.) (range 74–575) between the first exposure to OHP and the reaction.

Eight out of 124 (6.5%) patients reported only erythema and itching of the palms and flushing of the face and hands after the beginning of OHP infusion. Nine out of 124 (7.3%) patients developed a more severe reaction with dyspnoea, wheezing, laryngospasm, psico-motor agitation, tachycardia, precordial pain, diffuse erythema, itching and sweating. Only two patients experienced the symptoms at the end of the infusion, while the others developed the reaction between 10 to 15 min from the start of OHP infusion. All patients showing hypersensitivity were treated with steroids, many of them in association with antihistaminic drugs. The symptoms disappeared within half an hour to 2 h after stopping the OHP infusion and the beginning of the antiallergic therapy. One patient required hospitalisation for dyspnoea that disappeared in a few days.

Once the reaction had disappeared, nine patients continued the scheduled drug infusions, in particular 5-fluorouracile (5-FU) and Folinic acid, without any additional problem.

The percentage of reaction is different according to the chemotherapy regimens employed: 66.6% for OHP alone, 18.1% for FOLFOX-4 regimen, 8.8% for FOLFOX-3 regimen and 6.6% for OHP/CPT-11/c.i.5-FU/FA regimen (Table 2

**Table 2:** Number of reactions according to the regimen

**Chemotherapy regimen**	**No. of patients**	**No. of reactions**	**% reaction according to the regimen**	**Mean no. of infusions at the reaction onset**
FOLFOX-4	55	10	18.1	8.1
FOLFOX-3	34	3	8.8	14.3
Irinotecan Oxaliplatin Fuorouracil Folinic acid	30	2	6.6	12.5
Oxaliplatin	3	2	66.6	6

).

Three patients developed the reaction to the first chemotherapy treatment after a long period of rest. The total administered doses of OHP in patients developing the reaction are reported in Table 1. The cumulative dose of OHP was 1428 mg±176.7 (mean±s.e.) (range 360–2720 mg).

Six out of 17 patients with hypersensitivity reactions were successively re-exposed to OHP chemotherapy after premedication with steroids and antihistaminic drugs. Five of these six patients developed the same symptoms again, while one patient had no further reaction.

## DISCUSSION

Hypersensitivity reactions to platinum compounds are a well-known phenomena (Weiss, 1992). In the 1950s, literature reported the capacity of platinum salts to induce bronchial asthma among platinum-refinery workers (Hunter *et al*, 1945). It is not surprising that after the introduction of platinum compounds into chemotherapy, their association with type I hypersensitivity reactions was confirmed (Cleare *et al*, 1976). These reactions were first described for CDDP with a 5–20% incidence (Wiesenfeld *et al*, 1979; Shleback *et al*, 1995; Özgüroglu *et al*, 1999), and evidence regarding similar reactions for CBDCA are also available (Planner *et al*, 1991; Morgan *et al*, 1994; Weideman *et al*, 1994; Markman *et al*, 1999).

This kind of toxicity has been sporadically reported in clinical trials focusing on the effectiveness of OHP in chemotherapy or described as case reports (Machover *et al*, 1996; Diaz Rubio *et al*, 1998; Tournigand *et al*, 1998; Larzilliere *et al*, 1999; Medioni *et al*, 1999; De Gramont *et al*, 2000; Dold *et al*, 2002; Monnet *et al*, 2002).

Our results support the assumption that this side effect should not be underestimated. More than 13% of OHP-treated patients developed hypersensitivity reaction. This phenomenon is not well known, probably because OHP entered clinical practice only a few years ago. Moreover, according to our experience, the reactions generally develop after about 9–10 infusions. The relationship between the hypersensitivity reaction and OHP is supported by the following evidence. First, the symptoms developed a few minutes after starting the OPH infusion; secondly, the patients re-exposed to successive OHP administration developed a similar reaction; thirdly, two patients developed a reaction after monochemotherapy OHP infusion; finally, in patients treated with OHP/CPT-11/c.i. 5-FU/FA regimen, the reaction could be confused with a cholinergic syndrome due to CPT-11, but the responsibility of CPT-11 can be excluded since the re-exposure to CPT-11/c.i. 5-FU/FA without OHP was not able to provoke the hypersensitivity reaction.

The pathophysiology of hypersensitivity reactions is not clear, but the finding that almost all patients developed the reaction after multiple infusions of treatment suggests the need to be sensitised during previous cycles. Symptoms usually develop early after the start of the infusion and have been ascribed to a type I hypersensitivity Ig-E-mediated reaction (Stahl *et al*, 2001).

A different hypothesis suggests that platinum salts could induce an oligo or polyclonal T-cells expansion. These compounds can act as a superantigen on the peripheral blood mononuclear cells, thus releasing a large amount of proinflammatory cytokines (IL-6, TNF*α*, *γ* interferon) (Santini *et al*, 2001). The other possible mechanism consists in binding the platinum salts to different peptides of major histocompatibility complex (MHC).

In fact, HLA phenotype is a significant determinant of occupational sensitisation to inhaled hapten of complex platinum salts and the strength of this association varies according to the intensity of exposure (Newman Taylor *et al*, 1999).

Furthermore, the relationship between hypersensitivity reactions and HLA-haplotype has been described for other drugs (Hetherington *et al*, 2002). Additional factors are deemed to be necessary to the immune system for developing the reaction after several infusions.

Apart from hypersensitivity-related dyspnoea and wheezing, the lung may also be the target of a particular toxicity. A patient treated with OHP-5FU therapy developed severe dyspnoea. A bronchus alveolar lavage (BAL) and a lung biopsy diagnosed a diffuse alveolar damage that disappeared with steroid therapy (Trisolini *et al*, 2001).

In our experience, when a hypersensitivity reaction occurred, the infusion of OHP was immediately stopped and replaced by a saline infusion, an intravenous antihistaminic drug and a low-dose corticosteroids administration. In the case of more severe reactions (dyspnoea, sweating, bronchospasm, laryngospasm), we immediately administered a high dose of steroid. The steroid dose ranged between 100 and 1000 mg of hydrocortisone. After the reaction disappeared, the OHP infusion was not restarted and the decision to administer the other scheduled drugs was taken evaluating the clinical status of the patient after the reaction, the risk of additional toxicity and the clinical utility of the chemotherapy. In this way, about two-thirds of patients (11 patients) continued the infusion of other planned antiblastic drugs without any additional clinical problems.

In order to avoid further hypersensitivity problems in successive cycles, one can presumably explore a maximum prophylactic immunological blockage with a high dose of steroids and antihistaminic drugs for several days before the infusion of OHP, but the real benefit is uncertain because five out of six patients treated with steroids and/or antihistaminic drugs immediately before re-exposure developed the same intensity of reaction.

Documented data suggest that OHP as a continuous 6-h infusion seems to decrease the risk of hypersensitivity reactions. Only one out of 100 (1%) patients treated with OHP as a 6-h infusion added to chronomodulated 5-FU–FA as a first-line treatment of advanced colorectal cancer developed hypersensitivity-like reactions (Giacchetti *et al*, 2000). When OHP is infused in a chronomodulate setting (as a 12-h infusion) or flat infusion for 5 days, these hypersensitivity reactions do not occur. In particular, 151 patients submitted to 1087 constant rate continuous infusion courses of OHP, and 491 patients submitted to 3106 chronomodulate OHP courses did not experience any hypersensitivity reactions (Caussanel *et al*, 1990; Levi *et al*, 1992, 1993, 1994b, 1997, 1999; Bertheault-Cvitkovic *et al*, 1996).

Therefore, the incapacity of these schedules to produce hypersensitivity might be due to a long time infusion rather than to failure of activation of the immune system (which presents a circadian rhythm) in a chronomodulate setting (Levi *et al*, 1994a).

Interestingly, five patients who developed hypersensitivity reactions to 2-h OHP infusion, when re-exposed to 6-h OHP infusion, did not show any symptoms (Maindrault-Goebel *et al*, 2001). The mechanism is still unclear, although it is supposed that the maximum concentration reached by the drug is lower in a longer time of infusion. Theoretically this situation might occur with increased hydration, but no data are available. In the adjuvant setting, the number of allergic reactions is different from the advanced disease. In fact only 2% of the allergic, not already specified, reactions have been reported (De Gramont *et al*, 2002). The reason for this important difference is unclear.

It could be possible that the tumour releases factors able to make the immune system more sensitive, but no data are available.

In our study, neither the cumulative dose of OHP nor the lapse of time between the first exposition and the reaction are able to predict the hypersensitivity reaction. In our experience, the severity of clinical symptoms is variable and we cannot identify the patients at risk of developing the reaction or the factors indicating the patients where the reaction could be more severe. Particular attention is necessary when the lung is the target of reaction, because the re-exposure to OHP generally affects the same site with higher intensity. Special care is mandatory in patients receiving OHP for a long time and/or re-exposing to OHP after a pause.

In conclusion, a late hypersensitivity reaction seems a limiting toxicity of OHP administered in 2 h, and in patients previously affected it is advisable to avoid readministration of OHP with the same short schedule.

Literature data suggest that long-term OHP infusions are able to prevent the hypersensitivity reaction. The use of steroids does not seem useful in preventing hypersensitivity in patients having experienced previous reactions. On the contrary, long-term infusions, without dose decreasing, may prevent this reaction in patients previously affected, but a larger number of cases are required to provide definite responses on this matter.
